# The effect of music therapy on language communication and social skills in children with autism spectrum disorder: a systematic review and meta-analysis

**DOI:** 10.3389/fpsyg.2024.1336421

**Published:** 2024-05-07

**Authors:** Zijuan Shi, Si Wang, Maoqing Chen, Aimin Hu, Qingwen Long, Yujun Lee

**Affiliations:** ^1^North Sichuan Medical College, Nanchong, China; ^2^The Graduate School of Xi’an International Studies University, Shaanxi, China

**Keywords:** music therapy, autism spectrum disorder, children, language communication, social skill

## Abstract

**Background:**

Studies have shown that music therapy can be used as a therapeutic aid for clinical disorders. To evaluate the effects of music therapy (MT) on language communication and social skills in children with autism spectrum disorder (ASD), a meta-analysis was performed on eligible studies in this field.

**Methods:**

A systematic search was conducted in eight databases: PubMed, Embase, Web of Science, Cochrane Library databases, the China National Knowledge Infrastructure (CNKI), Wanfang Data, the Chinese Biomedical Literature (CBM) Database, and the VIP Chinese Science and Technology Periodicals Database. The standard mean difference (SMD) values were used to evaluate outcomes, and the pooled proportions and SMD with their 95% confidence intervals (CIs) were also calculated.

**Results:**

Eighteen randomized controlled trial (RCT) studies were included, with a total of 1,457 children with ASD. This meta-analysis revealed that music therapy improved their language communication [SMD = −1.20; 95%CI –1.45, −0.94; *χ*^2^ (17) = 84.17, *I*^2^ = 80%, *p* < 0.001] and social skills [SMD = −1. 13; 95%CI –1.49, −0.78; *χ*^2^ (17) = 162.53, *I*^2^ = 90%, *p* < 0.001]. In addition, behavior [SMD = −1.92; 94%CI –2.56, −1.28; *χ*^2^ (13) = 235.08, *I*^2^ = 95%, *p* < 0.001], sensory perception [SMD = −1.62; 95%CI –2.17, −1.08; *χ*^2^ (16) = 303.80, *I*^2^ = 95%, *p* < 0.001], self-help [SMD = −2. 14; 95%CI –3.17, −1.10; *χ*^2^ (6) = 173.07, *I*^2^ = 97%, *p* < 0.001] were all improved.

**Conclusion:**

Music therapy has a positive effect on the improvement of symptoms in children with ASD.

**Systematic review registration:**

https://www.crd.york.ac.uk/PROSPERO/.

## Introduction

Autism spectrum disorder (ASD) is a heterogeneous group of neurodevelopmental disorders characterized by difficulties in social communication, and abnormally limited behavior and interests, with a higher incidence in males than in females (Lai et al., 2014). Based on empirical data, approximately one in every 100 children worldwide is affected by Autism Spectrum Disorder (ASD), and this number has shown an upward trend over the past few decades (Zeidan et al., 2022). At present, there is no cure for ASD (Yang et al., 2020), its pathogenesis remains unclear (Dipasquale et al., 2017), and diagnostic methods are not satisfactory (Srikantha and Mohajeri, 2019). Currently, the treatment for children with autism primarily involves sensory integration training, behavioral and communication interventions, and educational training, among other interventions. Music therapy is a therapeutic approach that is now being increasingly applied in the field of healthcare (Foster et al., 2021). It is a therapeutic method that regulates the function of the cerebral cortex with melody, rhythm and harmony (Kasl-Godley and Gatz, 2000), and timbre and tone of music, and then changes the excitation and inhibition processes of the cerebral cortex, to improve social disorder and enhance the communication ability of autistic children (Srinivasan and Bhat, 2013). Because of the special significance of music therapy for autistic children, research in this field is being carried out both at home and abroad (Rabeyron et al., 2020; Yum et al., 2020; Chen et al., 2022; He Y. S. et al., 2022; He Y. et al., 2022).

Language function is a unique and advanced ability that is specific to humans (Bishop and Lerch, 2023). Human language function is specifically manifested in different forms such as listening, speaking, reading, and writing, and involves the involvement of different functional areas distributed across the cerebral cortex. These functional areas are closely interconnected through neural pathways (Schlosser et al., 2002). The language unit of syllable length and the human brain’s ability to synchronize neural firing rates are very important to children’s language acquisition (Kovelman et al., 2015). Language developmental disorders are the most common developmental disorders in children (Rißling et al., 2015). Among them, language development impairment caused by autism is one of the common types (Taghva and Mahabadi, 2013). Compared to typically developing children, children with autism have differences in brain connectivity, which may lead to language and communication difficulties (Lee et al., 2017). In addition, there is only a single channel to receive external information, which makes it difficult for autistic children to obtain the same learning and communication opportunities as typically developing child. A large number of cognitive neuropsychological research show that the cognitive/neural processing of language and music is not completely independent but with overlaps between the two, and a vast majority of neuroimaging research results show that language and music are closely related, with overlapping brain regions (Liu et al., 2022). Secondly, in terms of the social skills of autistic patients, autism’s social impairment is caused by a combination of factors, such as abnormal neurological development (Sokhadze et al., 2014), social cognition and theory of mind deficits (Yamada et al., 2022), emotional regulation difficulties (Yamada et al., 2022), as well as social anxiety and sensory hypersensitivity (McCullagh et al., 2020). Some researchers (Harms et al., 2010; Van der Donck et al., 2020) have indicated that the social impairments commonly observed in individuals with ASD may be associated with challenges in processing facial expressions. They have difficulty initiating social interactions and establishing interpersonal relationships with others. They lack interest in other people’s emotions and are unwilling to share their own interests with others (Barak and Feng, 2016). These factors work together to create challenges for individuals with autism in social interaction and language communication.

Music therapy, as a non-pharmacological treatment method, is gradually demonstrating its unique value in the treatment of ASD. Music can promote activity in brain regions associated with emotions and rewards, such as the ventral striatum and prefrontal cortex (Zatorre et al., 2001). Activation of these areas is particularly important for enhancing social motivation and emotional resonance (Mandic-Maravic et al., 2021). Furthermore, music therapy can activate and strengthen the brain’s mirror neuron system through engaging in musical activities (Kim et al., 2009; McCleery et al., 2013) (such as singing, playing instruments, etc.). The mirror neuron system plays a crucial role in imitation behavior, understanding others’ intentions and emotions, and social learning (Pavlenko et al., 2023). Additionally, music can also influence the brain’s language networks, including areas closely related to language processing such as Broca’s area and Wernicke’s area (Moreno, 2009). Music and language share neural processing mechanisms to some extent (Patel, 2003), and through music training, especially rhythm and melody training, progress can be made in language comprehension and production for individuals with autism.

In recent years, some researchers have conducted meta-analyses on the use of music therapy for treating ASD (Geretsegger et al., 2022; Ke et al., 2022). However, in contrast to these researchers, this study was performed to significantly verify the effect of music therapy on the language and social skills of children with ASD, in addition to conventional treatment. By searching randomized controlled trials (RCTs) on the effects of music therapy on the language and social skills of ASD children, we conducted a quantitative analysis using the meta method to evaluate the therapeutic effect of music therapy for autistic children and to provide further evidence-based medical evidence for music therapy in the field of autism treatment.

## Methods

We performed the systematic review with meta-analysis following the Preferred Reporting Items for Systematic Reviews and Meta-Analyses (PRISMA) (Page et al., 2021).

### Registration

The study was registered with PROSPERO (registration NO. CRD42023451556).

### Literature source

PubMed, Embase, Web of Science, Cochrane Library databases, the China National Knowledge Infrastructure (CNKI), Wanfang Data, the Chinese Biomedical Literature (CBM) Database, and the VIP Chinese Science and Technology Periodicals Database were searched up to May 17, 2023 for all eligible studies. Databases were searched for any articles with any combination of the following keywords in the Title, Abstract and Keywords: (1) ASD, autism, Autism Spectrum Disorder, Autistic (2) music therapy, music stimulation, music intervention, music training, melodic intonation therapy (3) children, school age, adolescence, youth, preschool. The specific search items were shown in the Supplementary material.

### Study selection

All eligible studies should have compared the effect of music therapy on language communication and social skills in ASD children. Studies were excluded if they met the following exclusion criteria: (1) duplicates; (2) reviews and/or meta-analyses; (3) case reports, letters, and conference summaries; (4) Non-musical therapy; (5) Outcomes did not report language communication or social skills; (6) Non-autistic children; (7) data duplication; (8) unavailable full texts; (9) guidelines, and comments; (10) experimental or animal studies; (11) important data missing; Neither publication language nor publication year was restricted.

### Data extraction

Data were extracted from each study: including first author, publication year, country, study design, type of publication, enrollment period, gender ratio, age, target population, and duration of treatment. The ABC (Aberrant Behavior Checklist) (Aman et al., 1985) and/or ATEC (Autism Treatment Evaluation Checklist) (Rimland and Edelson, 1999) scales were used to assess language communication, social skills, behavior, sensory perception, and self-help in children with ASD. The ABC scale encompasses various behavioral domains or sub-dimensions, such as sensory perception, social interaction, repetitive motor movements, language, and self-help, comprising a total of 57 items. Each item is rated on a scale of 1 to 4, with higher scores indicating more severe autistic behaviors. On the other hand, the ATEC scale consists of four self-assessment subscales: speech/language communication, social skills, sensory/cognitive awareness, and health/physical/behavior. The overall score on the ATEC scale ranges from 0 to 179, with higher scores indicating more pronounced symptoms of autism. In our study, we used the ABC scale to assess language communication, social skill, sensory perception, and self-help domains, and the ATEC scale to evaluate language communication, social skill, sensory perception, and behavior.

### Outcomes

The primary outcomes included language communication ability and social skills. The secondary outcomes included behaviors, sensory perception, and self-help.

### Definitions

The included subjects were children diagnosed with ASD in accordance with the Diagnostic and Statistical Manual of Mental Disorders (4th ed., DSM-4) (American Psychiatric Association, 1994) and the Diagnostic Statistical Manual of Mental Disorders (5th ed., DSM-5) (American Psychiatric Association, 2013). The music therapy included in this paper mainly includes music listening, social stories as the main content of music therapy, music teaching, and music games.

### Study quality

The quality of the RCT studies was assessed by two independent reviewers (Zijuan Shi and Yujun Lee) using the Cochrane Risk assessment tool (Higgins, 2011). This tool includes random sequence generation, blind methods for subjects and personnel, assignment concealment, selective reporting, incomplete outcome data, and other sources of bias. Judgement can be high risk, moderate risk, or low risk for each domain. In a study, an article is regarded as having a low risk of bias if the risk assessment for all domains is at low risk of bias; while one domain is at high risk of bias, then the article is rated as having a high risk of bias, and if a domain is at uncertain risk of bias, then the article is rated as having a moderate risk of bias.

### Statistical analyses

Meta-analyses were performed using the Review Manager 5.4 (Review Manager RevMan) [Computer program]. Version 5.4. The Cochrane Collaboration 2020 and STATA 15.0 (StataCorp LLC 4905 Lakeway Drive College Station, TX77845 United States). Inverse variance method was used with random effects model for data analysis. The pooled proportions and standard mean difference (SMD) with their 95% confidence intervals (CIs) were calculated to measure the effect size between two groups. We considered SMD of 0.2 a small effect size, SMD 0.5 a medium effect size and SMD 0.8 a large effect size (Cohen, 1992). Cochrane’s *Q* test and *I*^2^ statistics were adopted to assess heterogeneity among studies (Herrera et al., 2022), and *p* < 0.1 or *I*^2^ > 50% indicated statistically heterogeneity. To explore the sources of heterogeneity, subgroup meta-regression (Thompson and Higgins, 2002) and leave-one-out sensitivity analysis (Bown and Sutton, 2010) were performed by publication year (≥ 2020 vs. < 2020), study quality (low risk vs. moderate risk vs. high risk), duration of music therapy (>12 weeks vs. ≤12 weeks), age (>7 years old vs. ≤7 years old) and two different assessment tools (ATEC vs. ABC). If the number of included studies was greater than 2, subgroup analyses would be performed based on the covariates mentioned earlier. A *p*-value of less than 0.1 was considered as indicating a statistically significant interaction. Regression analysis was performed only when there were more than ten relevant studies (Higgins, 2011). Leave-one-out sensitivity analysis was performed by removing studies one by one from the meta-analysis. Publication bias was evaluated with the Egger’s test (Egger et al., 1997), and *p* < 0.1 indicated statistically significant publication bias.

## Results

### Study characteristics

**Figure 1:**
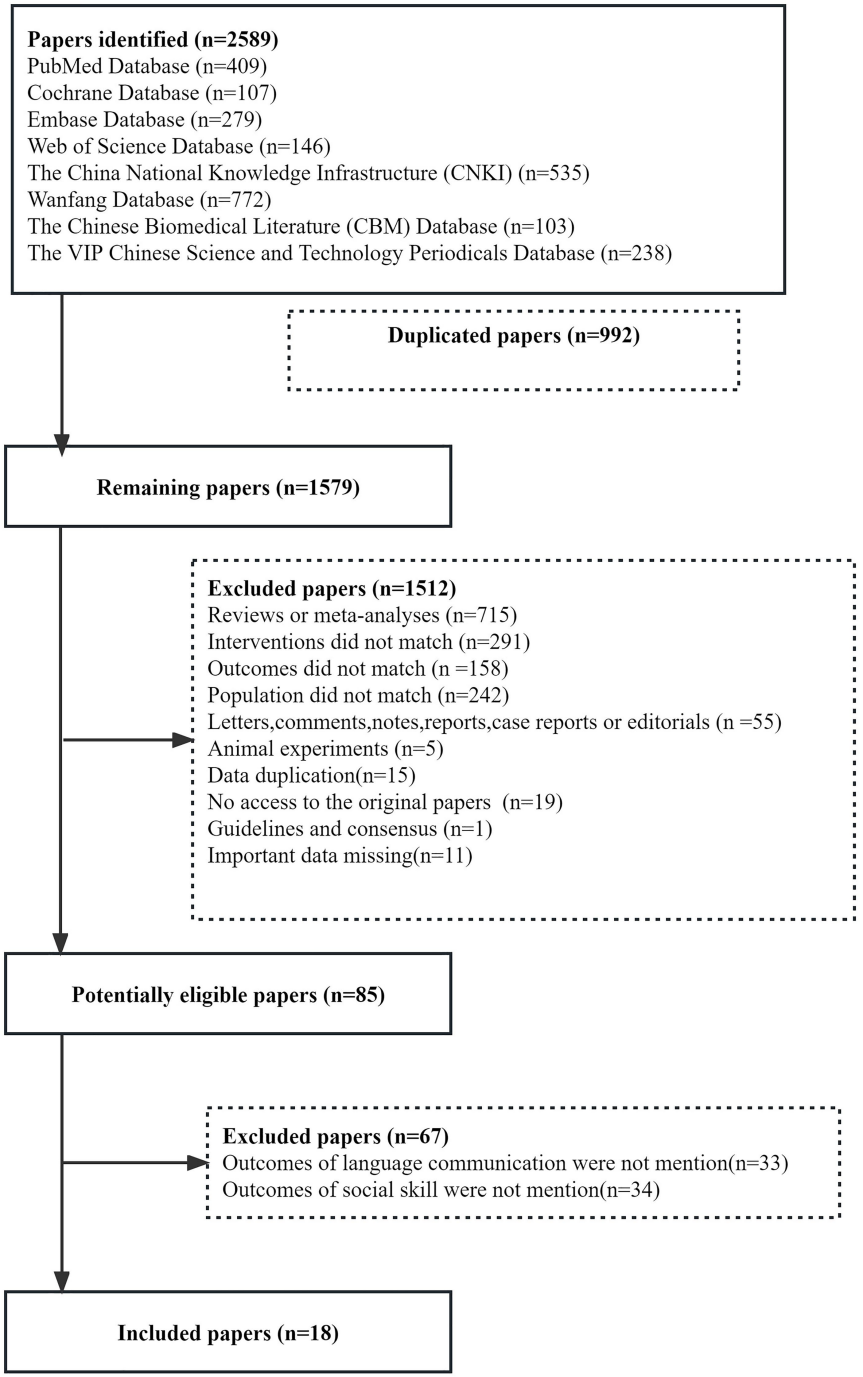
Flow chart of study selection.

**Figure 2:**
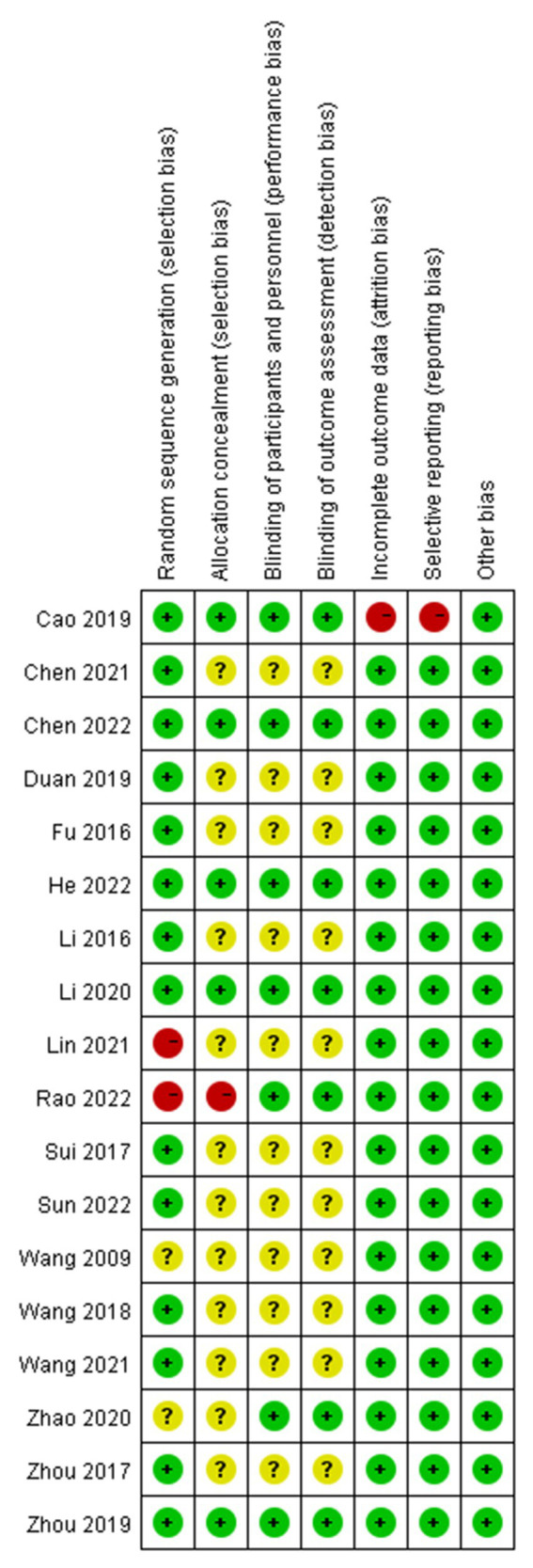
Risks of bias assessment of the included articles by each domain.

We retrieved 2589 studies from 8 databases, and after removing duplicates, two independent reviewersonducted screening and re-screening of titles and abstracts to gradually exclude irrelevant studies. A total ofeighteen studies (Wang et al., 2009; Fu et al., 2016; Li et al., 2016; Sui, 2017; Zhou et al., 2017; Wang, 2018; Cao, 2019; Duan, 2019; Zhou, 2019; Li, 2020; Zhao et al., 2020; Chen and Liu, 2021; Lin, 2021; Wang, 2021; Chen et al., 2022; He et al., 2022; Rao, 2022; Sun, 2022;) including 1,457 patients with ASD were finally included (Figure 1). Characteristics of the included studies are reported in Table 1. All studies are RCT studies with sample sizes ranging from 40 to 108 patients. All studies were published as full texts between 2009 and 2022. All studies were performed in Asia. Four studies (Zhou, 2019; Li, 2020; Chen et al., 2022; He et al., 2022) were of low risk, 11 studies (Wang et al., 2009; Fu et al., 2016; Li et al., 2016; Sui, 2017; Zhou et al., 2017; Wang, 2018; Duan, 2019; Zhao et al., 2020; Chen and Liu, 2021; Wang, 2021) were of moderate risk, and three studies were (Cao, 2019; Rao, 2022) of high risk (Figure 2).

**Table 1:** Characteristics of the included studies.

First author (year)	Country	Study design	Type of publication	Enrollment period	No. total Pts. (male, %)	Age (years)	Target population	Duration of disease (years)	Duration of treatment (weeks)	Language communication evaluation outcomes (Mean ± SD)	Social skills evaluation outcomes (Mean ± SD)	Sensory Perception evaluation outcomes (Mean ± SD)	Behavior evaluation outcomes (Mean ± SD)	Self-help evaluation outcomes (Mean ± SD)
Rao (2022)	CHN	RCT	Full-text	Jan 2020-Dec 2021	60 (57%)	MT:5.26 ± 1.38 Non-MT:5.24 ± 1.33	ASD	2.52 ± 0.72	5	MT:14.22 ± 2.66 (ABC) MT:13.02 ± 3.17 (ATEC) Non-MT:17.44 ± 3.34 (ABC) Non-MT:18.21 ± 3.23 (ATEC)	MT:8.14 ± 1.36 (ABC) MT:14.14 ± 3.64 (ATEC) Non-MT:10.25 ± 1.63 (ABC) Non-MT:19.39 ± 3.21 (ATEC)	MT:10.06 ± 2.11 (ABC) MT:14.44 ± 4.65 (ATEC) Non-MT:13.98 ± 2.22 (ABC) Non-MT:19.05 ± 4.14 (ATEC)	MT:14.21 ± 3.98 (ATEC) Non-MT:18.92 ± 4.06 (ATEC)	MT:5.87 ± 1.23 (ABC) Non-MT:8.02 ± 1.06 (ABC)
Chen et al. (2022)	CHN	RCT	Full-text	Jan 2019-Jun 2020	96 (56%)	MT:4.2 ± 1.1 Non-MT:3.9 ± 0.8	ASD	MT:1.6 ± 0.5 Non-MT:1.5 ± 0.3	24	MT:13.6 ± 1.8 (ABC) MT:15.6 ± 2.7 (ATEC) Non-MT:14.3 ± 1.5 (ABC) Non-MT:16.8 ± 2.9 (ATEC)	MT:14.4 ± 2.1 (ABC) MT:16.3 ± 2.4 (ATEC) Non-MT:15.3 ± 2.2 (ABC) Non-MT:17.3 ± 2.2 (ATEC)	MT:17.6 ± 2.2 (ABC) MT:10.2 ± 2.6 (ATEC) Non-MT:18.5 ± 1.9 (ABC) Non-MT:11.2 ± 2.1 (ATEC)	MT:14.3 ± 2. 8 (ATEC) Non-MT:15.4 ± 2.4 (ATEC)	MT:14.2 ± 1.2 (ABC) Non-MT:14.8 ± 1.4 (ABC)
Sun (2022)	CHN	RCT	Full-text	Jan 2020-Nov 2021	92 (55%)	MT:4.27 ± 1.41 Non-MT:4.23 ± 1.12	ASD	MT:2.10 ± 0.53 Non-MT:2.02 ± 0.45	12	MT:12.56 ± 2.41 (ATEC) Non-MT:16.42 ± 3.17 (ATEC)	MT:10.04 ± 1.87 (ATEC) Non-MT:15.65 ± 2.14 (ATEC)	MT:10.26 ± 1.85 (ABC) Non-MT:16.54 ± 1.31 (ABC)	MT:9.62 ± 1.04 (ATEC) Non-MT:14.31 ± 1.23 (ATEC)	NA
Chen and Liu (2021)	CHN	RCT	Full-text	Sep 2017-Dec 2020	100 (60%)	MT:4.46 ± 0.40 Non-MT:4.52 ± 0.39	ASD	MT:3.43 ± 0.65 Non-MT:3.41 ± 0.72	12	MT:15.20 ± 2.07 (ABC) MT:13.50 ± 1.50 (ATEC) Non-MT:17.89 ± 3.68 (ABC) Non-MT:18.09 ± 2.84 (ATEC)	MT:12.04 ± 2.39 (ABC) MT:14.37 ± 2.30 (ATEC) Non-MT:15.12 ± 2.64 (ABC) Non-MT:19.52 ± 3.14 (ATEC)	MT:4.31 ± 0.50 (ABC) MT:15.37 ± 1.83 (ATEC) Non-MT:7.48 ± 0.73 (ABC) Non-MT:20.10 ± 2.43 (ATEC)	MT:11.08 ± 1.32 (ATEC) Non-MT:16.30 ± 1.54 (ATEC)	MT:7.64 ± 1.08 (ABC) Non-MT:9.05 ± 1.13 (ABC)
Wang (2021)	CHN	RCT	Full-text	Jan 2018-Dec 2019	100 (51%)	MT:2.60 ± 0.48 Non-MT:2.55 ± 0.54	ASD	NA	24	MT:9.21 ± 3.76 (ATEC) Non-MT:13.01 ± 4.03 (ATEC)	MT:9.12 ± 4.48 (ATEC) Non-MT:14.00 ± 5.15 (ATEC)	MT:13.99 ± 4.24 (ATEC) Non-MT:20.03 ± 4.05 (ATEC)	MT:10.02 ± 3.97 (ATEC) Non-MT:14.01 ± 4.08 (ATEC)	NA
He (2022)	CHN	RCT	Full-text	Jan 2021-Oct 2021	100 (69%)	MT:3.66 ± 0.22 Non-MT:3.9 ± 0.18	ASD	NA	8	MT:18 ± 5 (ABC) Non-MT:20 ± 4 (ABC)	MT:13.5 ± 5.2 (ABC) Non-MT:14.1 ± 4.1 (ABC)	MT:11.80 ± 1.39 (ABC) Non-MT:11.02 ± 0.50 (ABC)	NA	MT:14.50 ± 1.56 (ABC) Non-MT:15.02 ± 0.95 (ABC)
Lin (2021)	CHN	RCT	Full-text	Jun 2020-Dec 2020	40 (63%)	MT:5.0 ± 1.0 Non-MT:5.1 ± 1.1	ASD	MT:2.1 ± 1.0 Non-MT:2.0 ± 1.3	12	MT: 12.5 ± 2.0 (ATEC) Non-MT:17.7 ± 2.8 (ATEC)	MT:10.2 ± 1.2 (ATEC) Non-MT:15.8 ± 1.7 (ATEC)	MT:9.8 ± 1.4 (ATEC) Non-MT:16.5 ± 2.6 (ATEC)	MT:9.3 ± 1.4 (ATEC) Non-MT:15.1 ± 1.9 (ATEC)	NA
Zhao et al. (2020)	CHN	RCT	Full-text	Jan 2016-Dec 2018	68 (63%)	MT:8.3 ± 4.0 Non-MT:8.6 ± 4.3	ASD	NA	15	MT:6.0 ± 3.3 (ABC) Non-MT:7.6 ± 3.4 (ABC)	MT:6.0 ± 3.3 (ABC) Non-MT:6.3 ± 4.2 (ABC)	NA	NA	NA
Li (2020)	CHN	RCT	Full-text	Sep 2016-Sep 2018	66 (71%)	MT:3.46 ± 1.02 Non-MT:3.33 ± 0.99	ASD	NA	24	MT:9.46 ± 3.83 (ATEC) Non-MT:12.54 ± 4.13 (ATEC)	MT:10.29 ± 3.56 (ATEC) Non-MT:13.33 ± 4.25 (ATEC)	MT:14.55 ± 3.14 (ATEC) Non-MT:19.33 ± 3.55 (ATEC)	MT:10.49 ± 4.27 (ATEC) Non-MT:13.25 ± 3.28 (ATEC)	NA
Zhou (2019)	CHN	RCT	Full-text	Dec 2017-May 2019	73 (78%)	MT:4.35 ± 1.46 Non-MT:5.52 ± 1.02	ASD	NA	8	MT:14.56 ± 1.27 (ABC) Non-MT:20.13 ± 3.51 (ABC)	MT:16.53 ± 6.84 (ABC) Non-MT:20.43 ± 8.23 (ABC)	MT:4.02 ± 1.21 (ABC) Non-MT:7.59 ± 3.28 (ABC)	NA	MT:12.32 ± 1.28 (ABC) Non-MT:18.96 ± 1.55 (ABC)
Duan (2019)	CHN	RCT	Full-text	Jan 2018-Dec 2018	90 (72%)	MT:5.3 ± 2.0 Non-MT:5.2 ± 2.2	ASD	MT:2.6 ± 0.5 Non-MT:2.5 ± 0.5	12	MT:12.0 ± 2.3 (ATEC) Non-MT:17.3 ± 2.8 (ATEC)	MT:10.5 ± 4.0 (ATEC) Non-MT:16.5 ± 3.8 (AEC)	MT:10.2 ± 1.6 (ATEC) Non-MT:16.8 ± 1.6 (ATEC)	MT:9.5 ± 1.2 (ATEC) Non-MT:14.6 ± 1.5 (ATEC)	NA
Cao (2019)	CHN	RCT	Full-text	Jun 2017-Jun 2018	80 (53%)	MT:5.32 ± 2.18 Non-MT:6.08 ± 2.74	ASD	NA	12	MT:12.6 ± 4.8 (ATEC) Non-MT:16.3 ± 3.5 (ATEC)	MT:9.85 ± 5.0 (ATEC) Non-MT:12.4 ± 3.2 (ATEC)	MT:10.1 ± 6.3 (ATEC) Non-MT:15.2 ± 3.1 (ATEC)	MT:10.7 ± 5.2 (ATEC) Non-MT:14.3 ± 3.0 (ATEC)	NA
Wang (2018)	CHN	RCT	Full-text	May 2015–May 2017	92 (71%)	MT:4.7 ± 3.1 Non-MT:5.2 ± 2.4	ASD	NA	NA	MT:12.71 ± 2.84 (ATEC) Non-MT:16.94 ± 4.26 (ATEC)	MT:12.53 ± 5.93 (ATEC) Non-MT:17.61 ± 6.15 (ATEC)	MT:15.24 ± 1.93 (ATEC) Non-MT:19.28 ± 3.41 (ATEC)	MT:16.12 ± 2.14 (ATEC) Non-MT:18.93 ± 2.08 (ATEC)	NA
Zhou et al. (2017)	CHN	RCT	Full-text	Sept 2013-Apr 2016	108 (77%)	MT:3.79 ± 1.12 Non-MT:3.72 ± 1.08	ASD	NA	24	MT:12.72 ± 2.41 (ABC) Non-MT:14.89 ± 2.62 (ABCs)	MT:18.09 ± 2.54 (ABC) Non-MT:19.13 ± 2.94 (ABC)	MT:10.13 ± 2.20 (ABC) Non-MT:11.55 ± 2.58 (ABC)	NA	MT:37.76 ± 5.75 (ABC) Non-MT:41.44 ± 6.68 (ABC)
Sui (2017)	CHN	RCT	Full-text	May 2015-Jun 2017	70 (57%)	MT:5.41 ± 1.77 Non-MT:5.36 ± 1.75	ASD	MT:1.08 ± 0.61 Non-MT:1.04 ± 0.58	12	MT:13.17 ± 1.26 (ABC) MT:9.46 ± 2.15 (ATEC) Non-MT:20.04 ± 2.04 (ABC) Non-MT:13.25 ± 3.05 (ATEC)	MT:14.27 ± 2.31 (ABC) MT:10.34 ± 1.98 (ATEC) Non-MT:20.85 ± 2.32 (ABC) Non-MT:13.21 ± 2.04 (ATEC)	MT:10.37 ± 2.16 (ABC) MT:13.25 ± 3.8 (ATEC) Non-MT:15.36 ± 3.06 (ABC) Non-MT:16.83 ± 3.9 (ATEC)	MT:10.21 ± 2.31 (ATEC) Non-MT:13.24 ± 3.05 (ATEC)	MT:8.73 ± 1.02 (ABC) Non-MT:16.83 ± 1.35 (ABC)
Fu et al. (2016)	CHN	RCT	Full-text	Feb 2014-Mar 2016	90 (47%)	MT:4.5 ± 0.5 Non-MT:3.5 ± 0.5	ASD	MT:1.5 ± 0.25 Non-MT:1.2 ± 0.5	24	MT:12.10 ± 4.36 (ATEC) Non-MT:17.01 ± 3.57 (ATEC)	MT:12.11 ± 6.01 (ATEC) Non-MT:16.31 ± 6.38 (ATEC)	MT:14.22 ± 2.39 (ATEC) Non-MT:19.85 ± 4.3 5 (ATEC)	MT:16.5 ± 2.4 (ATEC) Non-MT:19.2 ± 2. 2 (ATEC)	NA
Li et al. (2016)	CHN	RCT	Full-text	Dec 2012-Dec 2015	80 (73%)	MT:3.32 ± 1.23 Non-MT:3.45 ± 1.30	ASD	NA	10	MT:12.58 ± 2.31 (ATEC) Non-MT:16.43 ± 3.09 (ATEC)	MT:10.09 ± 1.98 (ATEC) Non-MT:15.43 ± 2.08 (ATEC)	MT:10.25 ± 1.87 (ATEC) Non-MT:16.41 ± 1.29 (ATEC)	MT:9.68 ± 1.01 (ATEC) Non-MT:14.32 ± 1.21 (ATEC)	NA
Wang et al. (2009)	CHN	RCT	Full-text	Feb 2005-Dec 2007	52 (31%)	Rang:(3–7)	ASD	NA	24	MT:12.09 ± 5.37 (ATEC) Non-MT:16.86 ± 6.12 (ATEC)	MT:12.26 ± 5.82 (ATEC) Non-MT:15.96 ± 5.36 (ATEC)	MT:14.67 ± 7.21 (ATEC) Non-MT:19.37 ± 7.51 (ATEC)	MT:16.18 ± 6.35 (ATEC) Non-MT:21.85 ± 7.65 (ATEC)	NA

CHN, China; RCT, randomized controlled trial; ASD, Autism Spectrum Disorder; MT, Music Therapy; ABC, Aberrant Behavior Checklist; ATEC, Autism Treatment Evaluation Checklist.

### Characteristics of patients

This study included 1,457 children (60% male). In the included studies, the children had an age range from 1.3 to 12.9 years old, with a mean (SD) age of 4.64 (1.29) years, and they were all diagnosed with ASD. Intervention durations ranged between 5 weeks and 24 weeks, with most interventions being 12 weeks or shorter. One study (Wang, 2018) did not report the intervention duration (*n* = 785/580/92, Table 1).

### Primary outcomes

#### Language

**Figure 3:**
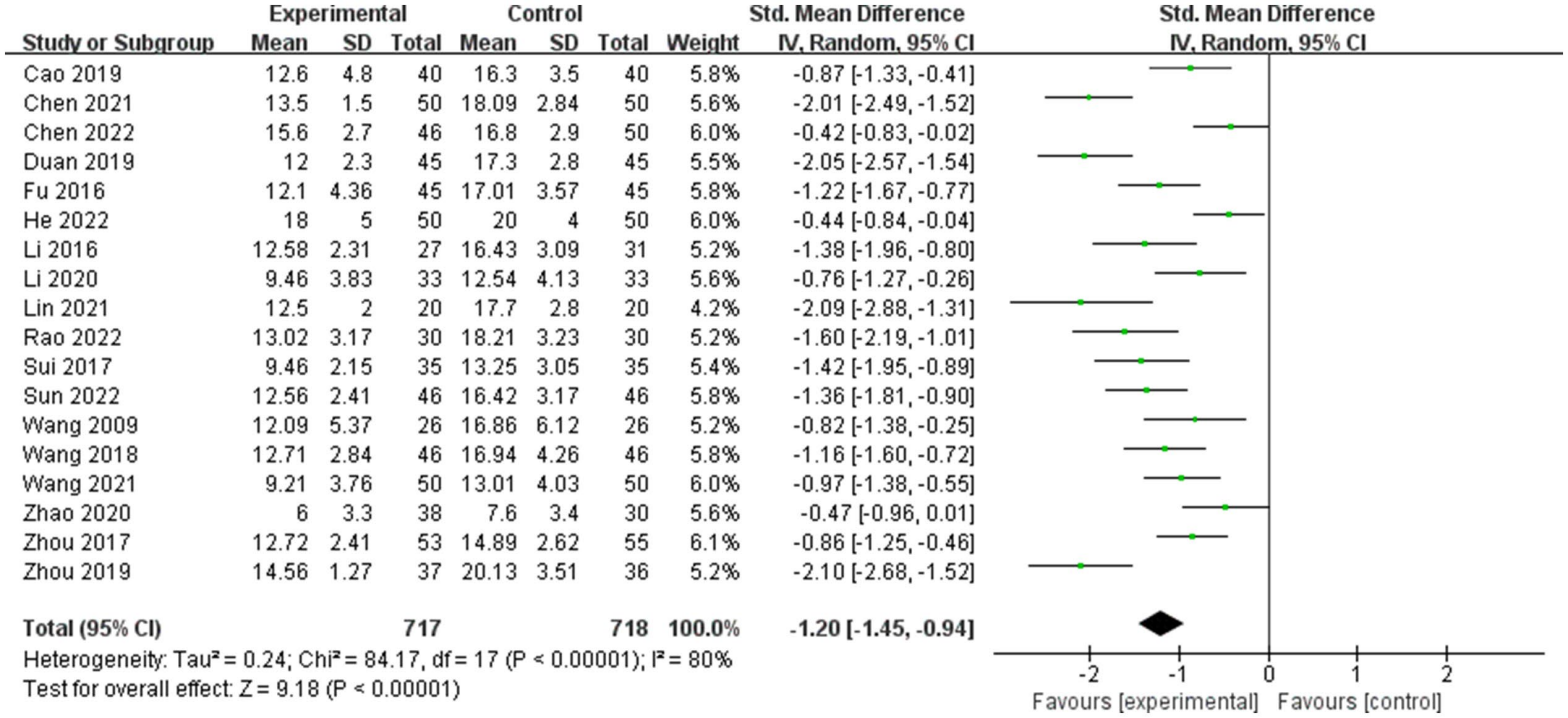
Forest map of the combined effective size for language communication.

Seventeen studies (Wang et al., 2009; Fu et al., 2016; Li et al., 2016; Sui, 2017; Zhou et al., 2017; Wang, 2018; Cao, 2019; Duan, 2019; Zhou, 2019; Li, 2020; Lin, 2021; Chen and Liu, 2021; Wang, 2021; Chen et al., 2022; He et al., 2022; Rao, 2022; Sun, 2022) reported that music therapy improved the communication ability of children with ASD. The combined effect size SMD was −1.20 (95%CI = −1.45, −0.94) (Figure 3), with significant heterogeneity between studies (*Q*-test: χ^2^(17) = 84.17, *p* < 0.001; *I*^2^ = 80%). Subgroup analysis showed that there was no significant difference observed between ABC scale and the ATEC scale (SMD: −1.29 vs. −1.27, Pinteraction = 0.95). Regarding different publication years, there was no significant difference observed between the language proficiency scores of papers published before 2020 and those published in 2020 and after (SMD: −1.30 vs. −1.09, Pinteraction = 0.41). From the perspective of paper quality, studies with low risk, moderate risk, and high risk had an SMD of −0.92, −1.25, and − 1.49 respectively, Pinteraction = 0.52. With respect to age, there was no significant difference observed between participants over 7 years old and those under 7 years old (SMD: −1.19 vs. −1.20, Pinteraction = 0.97). In terms of the duration of intervention, less than 12 weeks duration led to significantly higher scores than a duration greater than 12 weeks (SMD: −1.51 vs. −0.79, Pinteraction = 0.001) (Table 2). Meta-regression showed that different assessment tools (*p* = 0.016), paper quality (*p* = 0.001) and different publication years (*p* = 0.002) may be the sources of heterogeneity. A sensitivity analysis was conducted and suggested no source of heterogeneity. Egger’s test was performed as well, and the result showed significant publication bias between studies (*p* < 0.001).

**Table 2:** Subgroup analysis results for language communication.

(Language communication) groups	No.	SMD (95%CI)	Heterogeneity	*p*-value	Pinteraction
	studies		*I* ^2^		
Assessment tool					0.95
ATEC	14	−1.27 (−1.53, −1.00)	75%	<0.01	
ABC	8	−1.29 (−1.89, −0.69)	92%	<0.01	
Publication year					0.41
Before 2020	9	−1.30 (−1.61, −1.00)	70%	<0.01	
After 2020	9	−1.09 (−1.50, −0.68)	84%	<0.01	
Literature quality					0.52
High risk	3	−1.47 (−2.17,−0.77)	76%	<0.01	
Middle risk	11	−1.11 (−1.48, −0.75)	85%	<0.01	
Low risk	4	−0.91 (−1.58, −0.23)	88%	<0.01	
Intervention time					0.001
>12 weeks	7	−0.79 (−1.00, −0.58)	34%	0.17	
≤12 weeks	10	−1.51 (−1.89, −1.12)	82%	<0.01	
Age					0.97
>7	5	−1.19 (−1.69, −0.68)	82%	<0.01	
≤7	13	−1.20 (−1.51, −0.89)	81%	<0.01	

ABC, Aberrant Behavior Checklist; ATEC, Autism Treatment Evaluation Checklist; SMD, Standardized mean difference.

#### Social skills

**Figure 4:**
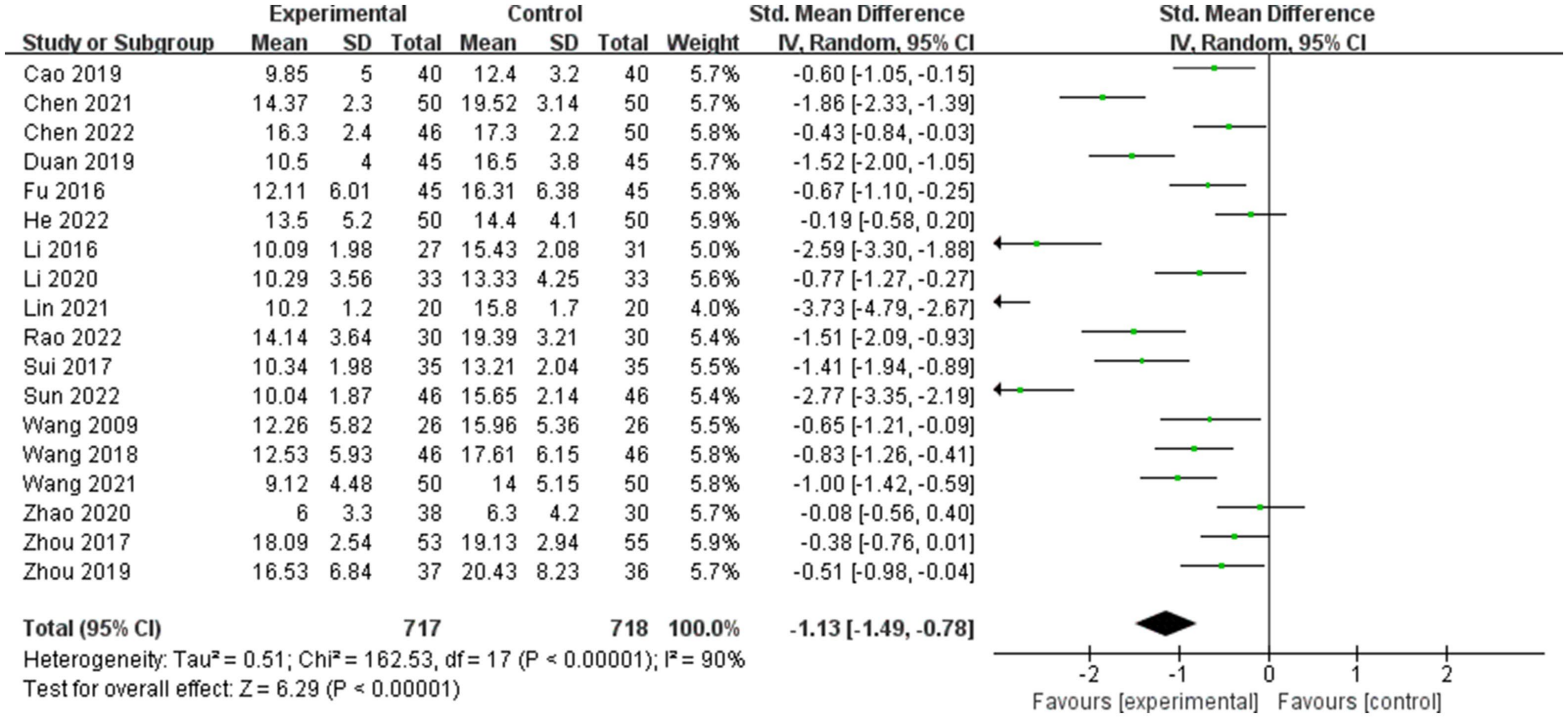
Forest map of the combined effective size for social skills.

Fifteen studies (Wang et al., 2009; Fu et al., 2016; Li et al., 2016; Sui, 2017; Wang, 2018; Cao, 2019; Duan, 2019; Zhou, 2019; Li, 2020; Chen and Liu, 2021; Lin, 2021; Wang, 2021; Chen et al., 2022; Rao, 2022; Sun, 2022) reported that music therapy improved the social skills of children with ASD. The combined effect size SMD was −1.13 (95%CI = −1.49, −0.78) (Figure 4), with significant heterogeneity between studies (*Q*-test: *χ*^2^(17) = 162.53, *p* < 0.001; *I*^2^ = 90%). Subgroup analysis showed that the social skill score in studies using ATEC was higher than that in studies using ABC (SMD: −1.39 vs. −0.81, Pinteraction = 0.07). In terms of different publication years, there was no significant difference observed between the social skill scores of papers published before 2020 and those published in 2020 and after (SMD: −1.31 vs. −0.98, Pinteraction = 0.39). From the perspective of literature quality, studies with low risk, moderate risk, and high risk had an (SMD of −0.44, −1.23, and −1.86 respectively, Pinteraction = 0.003). With respect to age, there was no significant difference observed between participants over 7 years old and those under 7 years old (SMD: −0.89 vs. −1.24, Pinteraction = 0.31). In terms of the duration of intervention, less than 12 weeks duration led to significantly higher scores than a duration greater than 12 weeks (SMD: −1.61 vs. −0.57, Pinteraction = 0.001) (Table 3). Meta-regression showed that different assessment tools (*p* = 0.003) and paper quality (*p* = 0.001) may be the sources of heterogeneity. The sensitivity analysis of these eighteen papers included in this study showed that none of them caused great interference to the results of this meta-analysis, suggesting that the results of this study were robust. There was significant publication bias between studies (*p* < 0.001).

**Table 3:** Subgroup analysis results for social skill.

(Social Skill) Groups	No.	SMD (95%CI)	Heterogeneity	*p*-value	Pinteraction
	studies		*I* ^2^		
Assessment tool					0.07
ATEC	14	−1.39 (−1.78, −0.99)	90%	<0.01	
ABC	8	−0.81 (−1.29, −0.32)	89%	<0.01	
Publication year					0.39
Before 2020	9	−0.98 (−1.36, −0.60)	82%	<0.01	
After 2020	9	−1.31 (−1.94, −0.68)	93%	<0.01	
Literature quality					0.003
High risk	3	−1.86 (−3.29, −0.44)	93%	<0.01	
Middle risk	11	−1.23 (−1.69, −0.77)	90%	<0.01	
Low risk	4	−0.44 (−0.67, −0.21)	8%	0.35	
Intervention time					0.001
>12 weeks	7	−0.57 (−0.79, −0.34)	43.0%	0.1	
≤12 weeks	10	−1.61 (−2.21, −1.02)	92%	<0.01	
Age					0.31
>7	5	−0.89 (−1.39, −0.38)	83%	<0.01	
≤7	13	−1.24 (−1.71, −0.78)	91%	<0.01	

ABC, Aberrant Behavior Checklist; ATEC, Autism Treatment Evaluation Checklist; SMD, Standardized mean difference.

### Secondary outcomes

#### Behavior

Fourteen studies (Wang et al., 2009; Fu et al., 2016; Li et al., 2016; Sui, 2017; Wang, 2018; Cao, 2019; Duan, 2019; Li, 2020; Chen and Liu, 2021; Lin, 2021; Wang, 2021; Chen et al., 2022; Rao, 2022; Sun, 2022) reported that music therapy improved the behavior of children with ASD. The combined effect size SMD was −1.92 (95%CI = −2.56, −1.28), with significant heterogeneity between studies (*Q*-test: *χ*^2^(13) = 235.08, *p* < 0.001; *I*^2^ = 94%). Subgroup analysis showed that there was no significant difference observed between the behavior scores of papers published before 2020 and those published in 2020 and after (SMD: −2.02 vs. −1.84, Pinteraction = 0.80). From the perspective of paper quality, studies with low risk, moderate risk, and high risk had an (SMD of −0.54, −2.30, and −1.71 respectively, Pinteraction <0.001). With respect to age, there was no significant difference observed between participants over 7 years old and those under 7 years old (SMD: −1.72 vs. −2.01, Pinteraction = 0.67). In terms of the duration of intervention, less than 12 weeks duration led to significantly higher scores than a duration greater than 12 weeks (SMD: −2.73 vs. −0.80, Pinteraction<0.001). Meta-regression analysis did not show the source of heterogeneity. The sensitivity analysis of these 14 papers included in this study showed that none of them caused great interference to the results of this meta-analysis, indicating that the results of this study were robust. There was significant publication bias between studies (*p* < 0.001).

#### Sensory perception

Sixteen studies (Wang et al., 2009; Fu et al., 2016; Li et al., 2016; Sui, 2017; Zhou et al., 2017; Wang, 2018; Cao, 2019; Duan, 2019; Zhou, 2019; Li, 2020; Chen and Liu, 2021; Lin, 2021; Wang, 2021; Chen et al., 2022; Rao, 2022; Sun, 2022) reported that music therapy improved the sensory perception of children with ASD. The combined effect size SMD was −1.62 (95%CI = −2.17, −1.08), with significant heterogeneity between studies (*Q*-test: *χ*^2^(16) = 303.80, *p* < 0.001; *I*^2^ = 95%). Subgroup analysis showed that there was no significant difference observed between ABC scale and the ATEC scale (SMD: −1.76 vs. −1.72, Pinteraction = 0.96). In terms of different publication years, here was no significant difference observed between the sensory perception scores of papers published before 2020 and those published in 2020 and after (SMD: −1.67 vs. −1.57, Pinteraction = 0.86). From the perspective of paper quality, studies with low risk, moderate risk, and high risk had an SMD of −0.36, −1.96, and −1.66 respectively, Pinteraction = 0.07. With respect to age, there was no significant difference observed between participants over 7 years old and those under 7 years old (SMD: −1.67 vs. −1.61, Pinteraction = 0.92). In terms of the duration of intervention, less than 12 weeks duration led to significantly higher scores than a duration greater than 12 weeks (SMD: −2.05 vs. −1.01, Pinteraction = 0.06). Meta-regression showed that different quality of paper (*p* = 0.018) may be the source of heterogeneity. The sensitivity analysis of these 17 papers included in this study showed that none of them caused great interference to the results of this meta-analysis, showing that the results of this study were robust. There was significant publication bias between studies (*p* < 0.001).

#### Self-help

Seven studies (Sui, 2017; Zhou et al., 2017; Zhou, 2019; Chen and Liu, 2021; Chen et al., 2022; He et al., 2022; Rao, 2022) reported that music therapy improved the self-help ability of children with ASD. The combined effect size SMD was −2.14 (95%CI = −3.17, −1.10), with significant heterogeneity between studies (*Q*-test: *χ*^2^(6) = 173.07, *p* < 0.001; *I*^2^ = 97%). Subgroup analysis showed that here was no significant difference observed between the self-help scores of papers published before 2020 and those published in 2020 and after (SMD: −3.94 vs. −0.96, Pinteraction = 0.13). From the perspective of paper quality evaluation, studies with low risk, moderate risk, and high risk had an SMD of −1.77, −2.72, and −1.85 respectively, Pinteraction = 0.72. With respect to age, there was a significant difference observed between participants over 7 years old and those under 7 years old (SMD: −6.7 vs. −1.45, Pinteraction<0.001). In terms of the duration of intervention, less than 12 weeks duration led to significantly higher scores than a duration greater than 12 weeks (SMD: −2.88 vs. −0.52, Pinteraction = 0.005). Due to the small number of the included studies that reported an effect of music therapy on the self-help ability of children with ASD, meta-regression and sensitivity analysis were not performed. There was significant publication bias between studies (*p* = <0.001).

## Discussion

The study population comprises children diagnosed with ASD and primarily focuses on individuals aged 13 and below. We included 18 independent studies, and through the synthesis and interpretation of these studies, our results suggest that combining music therapy with conventional treatment can effectively improve the therapeutic outcomes for children with ASD. Our research differs from previous meta-analyses as it focuses on two aspects of ASD: language communication and social skills deficits in individuals with ASD. Each study included in our analysis evaluated social and language impairments in children with autism spectrum disorder, with a total sample of 1,457 participants. The studies employed various music interventions, such as music therapy, music intervention, and music education. The primary outcome measure was the assessment of improvement in autism symptoms, which included not only social and language skills but also behavior, sensory perception, and self-care.

In terms of heterogeneity, the *I*^2^ values for the language communication and social skills were 80 and 90%, respectively, suggesting a high level of heterogeneity. After further analysis and exploration, as previously reported, we identified the sources of heterogeneity. The studies we included generally indicate that music therapy has a positive effect on the treatment of individuals with autism. However, we have also observed some variability in these effect sizes, which may be attributed to differences in study characteristics and specific intervention approaches. Indeed, we must acknowledge the potential presence of publication bias and selective reporting. Additionally, we conducted an evaluation of the reliability and applicability of the research findings, and overall, we identified a significant risk of bias. Music therapy is adopted to adjust and change people’s emotions and moods through feelings, experience, and understanding of music, improve people’s attention, memory, and imagination, and cultivate the perception of beauty and creativity. Music therapy is a non-drug treatment that is highly adaptable and flexible and directly benefits the patient physically, psychologically, and socio-emotionally (Kemper and Danhauer, 2005). Related studies also mentioned that ASD advocates comprehensive education and training, and music therapy is one of the auxiliary methods (Wang et al., 2019). Music learning for benefits autistic patients in that children are realized through the coupling of perception and movement and the adjustment of the sensorimotor network cortex during music learning or listening (Sharda et al., 2019). In addition, studies on relevant neural mechanisms have concluded that “music therapy can activate related brain areas regulate autonomic nerves, and regulate neurotransmitter release,” providing an objective basis for the clinical application of music therapy (Hao et al., 2023).

Regarding the enhancement of language proficiency, we found that music therapy enhances communication abilities by encouraging creative expression, fostering active participation, and facilitating emotional expression in individuals with autism. It is important to select appropriate music based on the child’s age, consider group or individual therapy depending on the situation, and pay attention to the timing of music therapy. The duration of treatment often yields different therapeutic effects, and extending the duration of music therapy can effectively enhance language communication abilities in children with autism. The intervention time should be at least three months (Shi et al., 2016), and in our study, the effectiveness of music intervention remained significant even with a duration of less than three months.

With regard to enhancing social skills, music can assist individuals with autism in expressing emotions, understanding the emotions of others, and engaging in nonverbal communication through sound, rhythm, and movement. It can also stimulate emotional responses and social interaction and facilitate communication with others. Music rhythm and melody can capture the attention of individuals with autism and increase their awareness of their surroundings. Additionally, music therapy can train nonverbal communication skills such as eye contact, body posture, and facial expressions. By providing a positive and interactive environment, music encourages active participation in social activities and enhances a sense of belonging within social groups, which ultimately improves social skills.

Although the focus of our meta-analysis was not centered around behavior, sensory perception, and self-care aspects, most of the literature also reported findings on these areas. Regarding behaviors in individuals with autism, the rhythm, melody, and harmony of music can stimulate the sensory system of the brain and provide positive and predictable sensory experiences to alleviate anxiety and stress, thus reducing the occurrence of maladaptive behaviors. Music therapy can also help individuals with autism reduce repetitive, stereotyped, and aggressive behaviors. In terms of sensory perception, music therapy can regulate the sensory system through appropriate auditory stimulation, helping patients better process and adapt to external stimuli. For example, gentle and harmonious music can create a soothing environment, alleviate hypersensitivity to sensory experiences, and improve attention and focus in individuals with autism. Finally, in terms of self-care, music therapy can assist individuals in learning and mastering self-care skills such as grooming, dressing, and eating through elements such as rhythm, lyrics, and movement. Music can serve as a heuristic tool, providing positive feedback and encouragement to motivate individuals with autism in developing their self-care abilities.

The study has several limitations which are mainly reflected in the following aspects: (1) Despite strict criteria for inclusion and exclusion of literature, the included forms of music therapy varied, and the duration, frequency, and intensity of interventions were also different. This variability may have an impact on the reliability of the results. (2) Music therapy is often combined with other conventional treatments, but there is a lack of professional music therapists who are familiar with medicine and psychology in China. Therefore, qualified music therapists cannot be guaranteed (Yu et al., 2021). (3) Currently, there is a lack of in-depth research on the specific mechanisms of the impact of music therapy on language and social impairments in children with autism. Further carefully designed studies are needed to reveal its exact effects. 4; Existing studies have issues such as small sample sizes, lack of control groups, or long-term follow-up, which require more rigorous research designs to evaluate the overall effectiveness and sustainability of music therapy. 5; Future research should further explore the optimal implementation of music therapy in the treatment of autism, including its specific benefits and synergistic effects with other intervention measures, in order to provide more comprehensive and evidence-based guidance for clinical practice.

In addition, although we searched Chinese and foreign databases, the treatment outcomes mainly focused on the improvement of language and social skills, and Chinese literature accounted for all the included studies. On the other hand, the assessment tools were relatively simple, and the quality assessment indicated that the quality of the included literature was not satisfactory. Therefore, a large number of high-quality literature at home and abroad will be needed in the future.

## Conclusion

In summary, music therapy can effectively improve the social skills and language communication ability of children with ASD, and enhance their behavioral ability, sensory perception, and self-care skills, having a positive effect on the improvement of the quality of life in children with ASD. Music therapy is worth promoting for early intervention in children with ASD. It is non-invasive, easy to implement, and suitable for the majority of ASD children.

Although our conclusion is that music therapy is effective, the specific intervention methods included in the literature vary. For example, there are differences in the selection of music for children of different age groups, the timing and duration of interventions, and the criteria for selecting professional music therapists. At the same time, research specifically focusing on language and social impairments in children with ASD under the context of music therapy is still limited. Further carefully designed studies are necessary to explore the exact mechanisms through which music therapy influences language development and social skills in this population. Lastly, it is necessary to conduct more rigorous research with larger sample sizes and control groups to determine the overall effectiveness and long-term sustainability of music therapy as a complementary intervention for ASD. These aspects are worth further investigation in future research.

In conclusion, although preliminary evidence suggests that music therapy has great potential in enhancing various functional domains, including social interaction and communication, in children with ASD, more comprehensive research is needed to determine the specific benefits and optimal implementation of music therapy in a broader context of ASD treatment.

## Data availability statement

The original contributions presented in the study are included in the article/Supplementary material, further inquiries can be directed to the corresponding author.

## Author contributions

ZS: Data curation, Formal analysis, Methodology, Resources, Software, Writing – original draft. SW: Data curation, Formal analysis, Resources, Writing – original draft. MC: Data curation, Writing – original draft, Methodology, Software. AH: Writing – original draft, Investigation, Resources, Validation. QL: Writing – original draft, Methodology, Project administration, Visualization. YL: Conceptualization, Funding acquisition, Supervision, Writing – review & editing.
