# Supplementary material for: The effect of music therapy on language communication and social skills in children with autism spectrum disorder: a systematic review and meta-analysis
Source: Front Psychol. 2024 May 7;15:1336421. doi: 10.3389/fpsyg.2024.1336421 (PMC11106491; doi:10.3389/fpsyg.2024.1336421)
Supplement: Supplementary file 1 [file Table_1.DOCX]

**Table S1** Strategies for database search

| **Database** | **search Strategy(May 2023)** |
| --- | --- |
| **Pubmed** | ((((((((music therapy[MeSH Terms]) OR (music,therapy)) OR (music)) OR (music intervention)) OR (music training)) OR (music stimulation)) OR (melodic intonation therapy)) AND ((((((((child[MeSH Terms]) OR (children)) OR (Youth*)) OR (school age)) OR (Adolescent)) OR (Adolescen*)) OR (Teen*)) OR (Preschool∗))) AND ((((((((((((((((Autism Spectrum Disorder[MeSH Terms]) OR (Autistic Spectrum Disorders)) OR (Disorder, Autistic Spectrum)) OR (Autism)) OR (autistic)) OR (ASD)) OR (Disorder, Autistic)) OR (Disorders, Autistic)) OR (Kanner's Syndrome)) OR (Kanner Syndrome)) OR (Kanners Syndrome)) OR (Autism, Infantile)) OR (Infantile Autism)) OR (Autism, Early Infantile)) OR (Early Infantile Autism)) OR (Infantile Autism, Early)) |
| **Embase** | ('autism'/exp OR 'autism spectrum disorder' OR 'autism, early infantile' OR 'autism, infantile' OR 'autistic child' OR 'autistic children' OR 'autistic disorder' OR 'autistic spectrum disorder' OR 'child development disorders, pervasive' OR 'childhood autism' OR 'classical autism' OR 'early infantile autism' OR 'infantile autism' OR 'infantile autism, early' OR 'kanner syndrome' OR 'pdd (pervasive developmental disorder)' OR 'pervasive child development disorders' OR 'pervasive developmental disorder' OR 'pervasive developmental disorders' OR 'typical autism') AND ('child':ab,ti OR 'children':ab,ti OR 'youth*':ab,ti OR 'school age':ab,ti OR 'adolescent':ab,ti OR 'adolescen*':ab,ti OR 'teen*':ab,ti OR 'preschool∗':ab,ti) AND ('music therapy':ab,ti OR 'music,therapy':ab,ti OR 'music':ab,ti OR 'music intervention':ab,ti OR 'music training':ab,ti OR 'music stimulation':ab,ti OR 'melodic intonation therapy':ab,ti) |
| **Cochrane** | ((music therapy) OR (music) OR (music intervention) OR (music training) OR (music stimulation) OR (melodic intonation therapy) ) AND ((child) OR (children) OR (Youth*) OR (school age) OR (Adolescent) OR (adolescen*) OR (Teen*) OR (preschool*) ) AND ((Autism Spectrum Disorder) OR (Autism Spectrum Disorders) OR (Disorder,Autistic Spectrum) OR (Autism) OR (Autistic) OR (ASD) OR (Disorder,Autistic) OR (Disorders,Autistic) OR (Kanner’s Syndrome) OR (Kanner Syndrome) OR (Kanners Syndrome) OR (Autism,Infantile) OR (Infantile Autism) OR (Autism,Early Infantile) OR(Early Infantile Autism) OR (Infantile Autism,Early) ) |
| **Web of Science** | (child OR children OR Youth* OR school age OR Adolescent OR Adolescen* OR Teen* OR Preschool∗) AND (Autism Spectrum Disorder OR Autistic Spectrum Disorders OR Autistic Spectrum Disorders OR Disorder, Autistic Spectrum OR Autism OR autistic OR ASD OR Disorder, Autistic OR Disorders, Autistic OR Kanner's Syndrome OR Kanner Syndrome OR Kanners Syndrome OR Autism, Infantile OR Infantile Autism OR Autism, Early Infantile OR Early Infantile Autism OR Infantile Autism, Early) AND (music therapy or music,therapy or music or music intervention or music training or music stimulation or melodic intonation therapy) |
| **CNKI**  **(China National Knowledge Infrastructure)** | (音乐疗法 + 音乐干预 + 音乐刺激 + 音乐护理 + 音乐训练 + 音乐治疗) * (自闭症 + 孤独症 + 阿斯伯格综合征 + 孤独谱系障碍 + 孤僻症) |
| **Wanfang Data** | (自闭症 or 孤独症 or 阿斯伯格综合征 or 孤独谱系障碍 or 孤僻症) and (音乐疗法 or 音乐干预 or 音乐刺激 or 音乐护理 or 音乐训练 or 音乐治疗) |
| **VIP**  **(VIP Chinese Science and Technology Periodicals Database)** | (M=(自闭症 OR 孤独症 OR 阿斯伯格综合征 OR 孤独谱系障碍 OR 孤僻症)) AND (M= (音乐疗法 OR 音乐干预 OR 音乐刺激 OR 音乐护理 OR 音乐训练 OR 音乐治疗)) |
| **CBM**  **(Chinese Biomedical Literature Database)** | ("音乐疗法"[常用字段:智能] OR "音乐干预"[常用字段:智能] OR "音乐刺激"[常用字段:智能] OR "音乐护理"[常用字段:智能] OR "音乐训练"[常用字段:智能] OR "音乐治疗"[常用字段:智能]) AND ("自闭症"[常用字段:智能] OR "孤独症"[常用字段:智能] OR "阿斯伯格综合征"[常用字段:智能] OR "孤独谱系障碍"[常用字段:智能] OR "孤僻症"[常用字段:智能]) |
